# Rethinking microglia from a circadian perspective in neuroimmunology: New insights

**DOI:** 10.4103/NRR.NRR-D-25-00157

**Published:** 2025-07-05

**Authors:** Daniele Mattei

**Affiliations:** Nash Family Department of Neuroscience, Icahn School of Medicine at Mount Sinai Hospital, New York, NY, USA

Microglia cells are the resident innate immune cells of the central nervous system (CNS) (Paolicelli et al., 2022). They play a pivotal role in CNS development and in maintaining homeostasis during adulthood. Microglia are being extensively studied for their involvement in CNS disorders, ranging from autoimmune diseases such as multiple sclerosis to neurodegenerative and psychiatric conditions, as well as stroke and brain tumors (Paolicelli et al., 2022). To harness microglia in therapeutic development, we need to deepen our understanding of their intricate biology. Our knowledge of microglia biology has evolved significantly with technological advancements, leading to a progressive “rethinking” of microglia cells within the field. Initially viewed as static cells, we now understand microglia to be highly motile and constantly surveillant. Once thought to be a homogeneous population of CNS macrophages, microglia are now recognized to occupy a range of cellular states (Paolicelli et al., 2022). Importantly, emerging evidence highlights circadian and diurnal rhythms as key contributors to microglial immune reactivity, morphology, and functions such as phagocytosis (Gu et al., 2023; Guzmán-Ruiz et al., 2023; Jiao et al., 2024). Circadian rhythms refer to physiological oscillations dictated by the endogenous biological clock, while diurnal rhythms refer to physiological variations set by external light cues that function as Zeitgeber time (ZT, German word for time-giver), such as the light-dark cycle (Guzmán-Ruiz et al., 2023). Currently, most microglial studies do not account for these oscillations, which can significantly impact study design and data interpretation. This perspective article aims to discuss why implementing a circadian framework in preclinical research is essential for advancing our understanding of microglia cells in both physiology and disease.

**Microglial circadian and diurnal variations:** Microglia express core clock genes such as *Bmal1* (*Arntl*), *REV-ERB* (*Nr1D1*), *Per1-2*, *Cry1-2*, and *Clock*. Pioneering work by Dr. Laura Fonken, Dr. Erik Musiek, and others, has shown that clock genes, such as *Bmal1* and *REV-ERB*, regulate microglial morphology, metabolism, immune response, and phagocytic functions (reviewed in: Guzmán-Ruiz et al., 2023; Jiao et al., 2024). Two studies have confirmed a diurnal variation in microglial morphology and process motility which results in different levels of microglial contact with synapses during wake and sleep states (Gu et al., 2023; Guzmán-Ruiz et al., 2023). Accordingly, it has been shown that microglial engulfment of synapses is subject to diurnal variations (Guzmán-Ruiz et al., 2023; Jiao et al., 2024; Mattei et al., 2024). Importantly, markers of microglial activation and phagocytosis, such as IBA1 and CD68, also exhibit diurnal expression patterns, along with the expression of cytokines (Guzmán-Ruiz et al., 2023; Jiao et al., 2024).

Changes in microglia morphology, surveillance, and immune functions between wake and sleep phases suggest that a transcriptional reprogramming occurs between the light and dark phases. Only recently has the microglial transcriptome been examined from a diurnal and circadian perspective using whole-genome RNA sequencing. We conducted a comparative RNA-sequencing analysis of adult mouse hippocampal microglia isolated at ZT4 (4-hour post light-off, wake phase) and ZT16 (4-hour post light-on, sleep phase), identifying significant expression differences in genes associated with immune response, immunometabolism, motility, and phagocytosis. Proteomic analysis confirmed similar changes also at the protein level. Notably, disease-associated microglia genes, such as *Crybb1* and *Spp1*, as well as homeostatic genes *Tmem119*, *P2ry12*, and *Cx3cr1*, displayed distinct expression profiles between light and dark phases (Mattei et al., 2024). Functionally, these changes correlated with increased microglial synaptic engulfment during the wake phase and enhanced response to systemic lipopolysaccharide during the sleep phase. Additionally, circular RNAs, a key class of regulatory non-coding RNAs, exhibited differential expression between phases in hippocampal microglia, suggesting their involvement in diurnal transcriptional reorganization (Ivanov et al., 2022).

Two recent studies provided further confirmation and in-depth analysis of circadian and diurnal oscillations in the microglial transcriptome. Sheehan et al. (2024) investigated the circadian transcriptional oscillations in cortical microglia from adult mice kept under constant darkness. The latter protocol is used to discern intrinsic circadian changes from diurnal changes dictated by external light cues. To this aim, following standard 12-hour:12-hour light:dark entrainment, mice are placed in constant darkness for 24 hours prior to and during sample collection. Sheehan et al. (2024) collected microglia every 2 hours across 24 hours and identified over 4000 rhythmic genes associated with immunometabolism, lysosomal functions, and immune signaling pathways. Interestingly, they also found disease-associated microglia genes to display a rhythmic expression (Sheehan et al., 2024). Conversely, Ouk et al. (2024) collected whole brain microglia for RNA sequencing every 4 hours across 24 hours from mice kept on a standard light:dark cycle and identified 1901 diurnally rhythmic microglial genes. Because the animals were not kept in constant darkness, the transcriptional fluctuations observed by Ouk et al. (2024) likely reflect a combination of intrinsic circadian and extrinsic diurnal influences on microglial regulation. While this approach offers a broader view of transcriptional rhythmicity, it does not allow for a clear distinction between changes driven by external light cues and those governed by the internal circadian clock. These data are the first evidence of the microglial circadian and diurnal transcriptional oscillations that likely underlies functional changes in immune response, morphology, motility, and phagocytosis between phases (Guzmán-Ruiz et al., 2023).

**Integrating microglial circadian and diurnal dynamics into preclinical research:** Most published preclinical studies investigating microglia simply state that animals are maintained on a 12-hour:12-hour light:dark cycle. However, they rarely specify the exact light-on/off schedule or the ZT of sample collection. Circadian and diurnal oscillations in transcriptome and proteome across 24 hours can lead to within- and between study variability in microglial readouts due to different ZT of sample collection. This is particularly relevant at a time when microglial multi-omics characterizations are increasing through single-cell RNA sequencing, spatial transcriptomics, and proteomic approaches. Accounting for ZT of sample collection can increase reproducibility of findings, when differences are due to diurnal and circadian factors.

**Figure 1:**
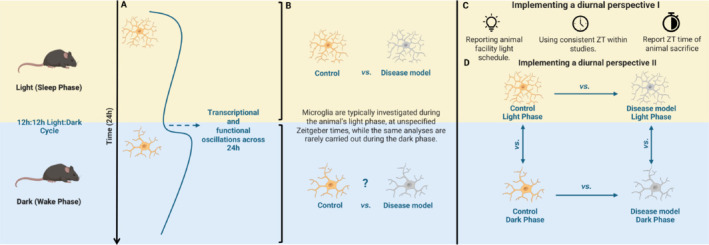
Investigating microglia cells across the light-dark cycle. (A) Emerging preclinical evidence demonstrates that microglia exhibit transcriptional and functional oscillations across 24 hours. (B) Rodents are typically housed in facilities with a 12-hour light:dark cycle, and most experiments are conducted during the animals’ sleep (light phase). Consequently, microglial features in physiology and disease are studied at unspecified Zeitgeber times (ZTs) during the light phase, while similar analyses during the dark phase remain largely unexplored. (C) Initial steps to account for microglial diurnal variations include reporting the light schedule of animal facilities and the ZT of sample collection, as well as maintaining consistent ZT sampling within and across studies. (D) A further step involves collecting samples at one time point in both the light and dark phases, ensuring the same number of hours post light-on and light-off. This design enables between-group comparisons of microglial readouts during both phases and within-group comparisons to assess differences between light and dark phases under control conditions and in disease states. To facilitate sampling during both phases, animal facilities can include rooms with a 12-hour dark:light cycle alongside the standard light:dark cycle. After a 14-day acclimatization period, animals housed under both cycles in parallel can simplify the implementation of this study design, providing a practical way to account for microglial diurnal variations in preclinical research. Created with BioRender.com.

Beyond enhancing reproducibility, accounting for microglial circadian and diurnal oscillations can significantly advance our understanding of their roles in physiology and disease. Current immune-pharmacological strategies rely on data from one phase only, usually, the light (sleep phase), as most animal facilities are set on a 12-hour:12-hour light:dark cycle, and rodents are nocturnal animals. Expanding analyses to include additional time points is likely to yield deeper insights into microglial involvement in disease (**Figure 1A** and **B**). Notably, Sheehan et al. (2024) demonstrated that the microglial circadian rhythmicity is impaired in aged mice as well as in the APP/PS1 model of Alzheimer’s disease. Similarly, Ouk et al. (2024) showed that microglia displayed compromised diurnal transcriptional oscillations in a mouse model of Huntington’s disease. These findings suggest that only relying on a single transcriptional snapshot during the sleep phase might be reductive. While not all research groups have the resources to embark on full circadian characterizations, a practical alternative is sampling microglia at corresponding time points in both light and dark phases as described in **Figure 1**. For example, if microglia are isolated 4 hours post light-on (ZT4) during the sleep phase for downstream analyses, what would the same analysis reveal if carried out also 4 hours post light-off (ZT16) during the wake phase? We recently demonstrated that in a maternal immune activation model of neurodevelopmental disorders, the nature of microglial transcriptional changes depends on whether cells are isolated during the sleep or wake phase, with higher enrichment of pro-inflammatory pathways present in microglia isolated at ZT18 compared to ZT6 (Mattei et al., 2024). Similarly, Sheehan et al. (2024) showed that disease-associated transcriptional signatures in microglia from APP/PS1 mice differ depending on whether isolation occurs in the morning or evening. In line with this, Ni et al. (2019) found that microglia from the APP-KI model of Alzheimer’s disease display greater enrichment of pro-inflammatory transcripts at ZT14 compared to ZT2. These findings highlight the importance of ZT in experimental design, as specific genes and pathways may only be detectably altered during distinct windows across the light–dark cycle. In turn, this can have important implications when looking for pharmacological targets aimed at modulating microglial molecular and functional states.

**Practical considerations:** **Figure 1C** outlines a few initial steps investigators can take to begin incorporating circadian and diurnal features into microglia research. These include reporting the animal facility’s light-on/off schedule as well as the ZT of sample collection. Additionally, maintaining consistency in ZT across studies for sample collection is important. A more advanced step, shown in **Figure 1D** and proposed in our recent work (Mattei et al., 2024), involves collecting samples at a single time point in both the light and dark phases. This approach enables a standard between-group comparison of microglial readouts (e.g., gene expression, protein levels, or functional assays) across phases, while ensuring the same number of hours following light-on and light-off. Such a design also facilitates within-group comparisons, allowing investigators to study differences between phases in control conditions (e.g., control light-phase *vs.* control dark-phase, **Figure 1D**) and to examine how these differences are altered in disease (e.g., disease light-phase vs. disease dark-phase, **Figure 1D**). This approach has its limitations, as taking a single snapshot of microglia at one time point in each phase from rodents not kept under constant darkness does not allow investigators to distinguish changes driven by external light cues from those governed by the intrinsic clock. However, it provides a broad overview of potential differences between the two phases in physiological conditions and disease.

A practical way to implement this design is to organize an animal facility with rooms set to a 12-hour:12-hour dark/light cycle, hosting animals under opposite schedules in parallel. Following a 14-day acclimatization period, this setup allows for simultaneous sampling in both phases, avoiding inconvenient collection times. This design raises important considerations, such as determining the most appropriate ZT for sample collection, given that the microglial transcriptome and function oscillate throughout the 24-hour cycle. Sheenhan et al. (2024) demonstrated that the peak expression times of microglial circadian genes are broadly distributed throughout the 24 hours, while Ouk et al. (2024) found a biphasic microglial diurnal (i.e., in mice not kept in constant darkness) transcriptional variation corresponding to dusk and dawn. Consequently, these data can be used as a reference to select specific ZT for microglial sampling based on the specific research question. Ultimately, implementing this simple approach would bridge a key biological feature of microglia cells to common preclinical research, potentially advancing our understanding of the microglial states and functions in health and disease. Incorporating a circadian perspective in microglia research holds significant translational promise. By uncovering the temporal dynamics of microglial state transitions and reactivity across physiological and pathological conditions, this approach may refine our understanding of disease progression and therapeutic windows. Ultimately, this could inform the development of chronopharmacological strategies aimed at optimizing treatment efficacy by aligning interventions with the microglial molecular and functional rhythms.

A challenge in this context is how to introduce a circadian/diurnal perspective in human microglia research, given that most freshly isolated microglia samples are derived from fresh and frozen postmortem brain tissue. As the field advances in implementing a circadian perspective in preclinical settings, it is essential to discuss how it can be applied to human research, where it has already been shown that peripheral immune cells also display diurnal transcriptional oscillations (Kervezee et al., 2018; Manella et al., 2022). One potential strategy is to incorporate time-of-death and clock gene expression data to stratify postmortem microglial samples into defined day and night windows, while carefully controlling for postmortem interval effects. Furthermore, human induced pluripotent stem cell-derived microglia offer a promising platform to model circadian dynamics *in vitro*. Circadian entrainment can be induced using established protocols such as serum shock or timed glucocorticoid treatments, which synchronize cellular clocks across populations (Pavan et al., 2022). These models could allow researchers to dissect the molecular and functional oscillations of human microglia, providing an entry point to study temporal regulation in disease-relevant genetic backgrounds and response to pharmacological stimuli.
